# Alterations of JNK Signaling Pathway Activity in the Rat Retina: Effects of Age, Age-Related Macular Degeneration-like Pathology, and a JNK Inhibitor (IQ-1S)

**DOI:** 10.3390/cells14120896

**Published:** 2025-06-13

**Authors:** Natalia A. Muraleva, Dmitry I. Tikhonov, Anna A. Zhdankina, Mark B. Plotnikov, Andrei I. Khlebnikov, Sergey V. Logvinov, Nataliya G. Kolosova

**Affiliations:** 1 Federal Research Center Institute of Cytology and Genetics SB RAS, 10 Pr. Akad. Lavrentieva, Novosibirsk 630090, Russia; 2 Department of Histology, Embryology and Cytology, Siberian State Medical University, Tomsk 634055, Russia; 3 Department of Pharmacology, Goldberg Research Institute of Pharmacology and Regenerative Medicine, Tomsk NRMC, 3 Lenin Ave., Tomsk 634028, Russia; 4 Radoiphysical Faculty, National Research Tomsk State University, Tomsk 634050, Russia; 5 Kizhner Research Center, Tomsk Polytechnic University, 30 Lenin Ave, Tomsk 634050, Russia

**Keywords:** age, retina, age-related macular degeneration, JNK signaling, synapses, JNK inhibitor, IQ-1S, OXYS rats

## Abstract

Age-related macular degeneration (AMD) is the leading cause of irreversible visual impairment worldwide. AMD development is associated with inflammation, oxidative stress, and a progressive proteostasis imbalance, in whose regulation, c-Jun N-terminal kinases (JNKs) play a crucial role. JNK inhibition is being discussed as a new way to prevent and treat AMD, but there are no data on JNK signaling in the retina and its changes with age and with AMD development. Here, for the first time, we assessed JNK-signaling activity in the retina and did not detect its age-related changes in healthy Wistar rats. By contrast, manifestation and progression of the AMD-like pathology in OXYS rats occurred simultaneously with JNK pathway activation. We also confirmed that selective JNK3 inhibitor 11H-indeno[1,2-b]quinoxalin-11-one oxime sodium salt (IQ-1S) can suppress neurodegenerative changes in the OXYS rat retina. Its effects were prevention of the destructive changes in retinal synapses and the suppression of the JNK signaling pathway activity during active progression of AMD signs in OXYS rats.

## 1. Introduction

Age-related macular degeneration (AMD) is a complex, multifactorial neurodegenerative disease that is the leading cause of irreversible vision loss in people over 60 years of age. Interaction of numerous genetic and environmental factors has an impact on this disease’s development, but age is the most significant risk factor for AMD; its incidence is increasing with increasing life expectancy. This disease is classified into dry (atrophic) AMD and wet AMD, depending on the presence of choroidal neovascularization. Most AMD starts as the dry type, and in 10–20% of individuals, it progresses to the wet type. Although the advent of antiangiogenesis therapy has helped to prevent blindness and restore vision in wet AMD, there remains no effective treatment for patients with the dry type (~90% of all AMD cases) [1,2]. With aging, the retina undergoes cell type-specific structural and functional changes that contribute to the development of AMD. The typical (for aging) decline of the choroid, of the retinal pigment epithelium (RPE), and of Bruch’s membrane underlie AMD pathogenesis, but mechanisms that trigger the transition from normal age-related alterations to the disease are unclear, thereby raising the question of what a normal aged retina is and what a diseased aged retina is. At the cellular level, changes that occur in the aged retina parallel those documented in the brain: both neuronal cell bodies and axons are damaged, the number of synapses decreases, dendrite architecture is remodeled, and the transmission of nerve impulses is compromised [3]. It has been found that key presynaptic components of individual photoreceptor ribbon synapses remain stable throughout life, but there is a significant loss of synapses in the outer plexiform layer (OPL) of the aged mouse retina [4].

Synaptic dysfunction is a key hallmark of many neurodegenerative diseases; in particular, AMD and Alzheimer’s disease (AD) both show synaptic dysfunction, with synaptic loss preceding the loss of neuronal cell bodies [5]. Although age-related neurodegenerative disorders have many different pathological manifestations and causes acting through different intracellular molecular pathways, a number of studies strongly support the notion that c-Jun N-terminal kinases (JNKs) are a central actor in the degenerative mechanisms (affecting synapses) that characterize both neurodevelopmental and neurodegenerative diseases [6]. Moreover, the development of AMD is associated with inflammation, oxidative stress, and a progressive proteostasis imbalance, in the regulation of which JNK proteins play a crucial role [7]. Sustained activation of a JNK leads to synaptic dysfunction and even neuronal apoptosis, ultimately resulting in memory deficits and neurodegeneration [7]. Disturbances of signaling in JNK-controlled pathways are observed in the development of various neurodegenerative diseases [6,7]. As studies on various retinal-cell cultures and animal models indicate, JNK signaling may contribute to the pathogenesis of AMD [8]. Accordingly, pharmacological modulation of JNK signaling is being discussed as a potential target for the prevention and treatment of AMD and other neurodegenerative diseases [9].

Three genes encode three isoforms of the protein: JNK1, JNK2, and JNK3. JNK1 and JNK2 are expressed ubiquitously throughout the body, while JNK3 expression is seen mainly in the brain, heart, and testicles [10]. Currently, there is an active search for JNK3 inhibitors [11,12,13], some of which have already confirmed neuroprotective activity in animal models [14]. One of them is a selective JNK3 inhibitor called IQ-1S (sodium salt of 11H-indeno[1,2-b]quinoxalin-11-one oxime) [15], which, as shown in in silico and in vitro experiments, has strong affinity for JNK3, protects the brain after stroke in rats [16], and has anti-inflammatory properties [17]. We recently evaluated the neuroprotective potential of IQ-1S using senescence-accelerated OXYS rats, which spontaneously develop a phenotype similar to human age-related disorders including retinopathy (similar to AMD) and key features of AD. We showed that IQ-1S effectively suppresses the development of neurodegenerative processes in the brain [18,19] and retina [20] of OXYS rats; however, effects of IQ-1S on the level of JNK3 and on the activity of the JNK pathway in the retina were not assessed, and neither were resultant retinal-synapse/synaptic-density changes.

Age-related macular degeneration is a multifactorial disease involving various complex genetic and environmental factors. Reflecting this complexity, there is currently no single animal model that develops all signs of AMD in a progressive manner. The retinopathy that develops in OXYS rats corresponds to the dry type of AMD and manifests itself as signs of this disease: dystrophic changes and thinning of the retina, impaired microcirculation in the choroid, alterations of neurotrophic supply, accumulation of lipofuscin and amyloid β, and structural characteristics of AMD [21]. First clinical manifestations of retinopathy are detectable during ophthalmoscopic examination in ~20% of OXYS rats at 5–6 weeks of age, and at the age of 3–4 months, signs of retinopathy are present in all animals. The pathological changes progress and reach pronounced stages, implying a loss or significant deterioration in visual acuity, by age 14–18 months.

In the present study, for the first time, we examined the age-related changes in the JNK signaling activity in the retina of healthy Wistar rats and the contributions of this activity’s alteration to the development of the AMD-like pathology in OXYS rats, including the preclinical stage. As an indicator of the signaling pathway activity, we used the alteration of phosphorylation of a key signaling pathway kinase (JNK3) and of its target proteins: amyloid β precursor protein (APP) and c-Jun, which are the main downstream markers for this pathway [12,13].

We also assessed the relation between the previously identified retinoprotective effect of IQ-1S and its influence on the activity of the JNK3 signaling pathway and on preservation of synapses during active progression of AMD signs in OXYS rats (from age 4.5 months to 6 months).

## 2. Materials and Methods

### 2.1. Animals

Senescence-accelerated OXYS rats and Wistar rats were obtained from the Breeding Experimental Animal Laboratory of the Institute of Cytology and Genetics, the Siberian Branch of the Russian Academy of Sciences (Novosibirsk, Russia). The animals were housed under standard laboratory conditions (22 ± 2 °C and 12 h light/dark cycle) in cages (57 × 36 × 20 cm) with five animals in each, with ad libitum access to standard feed for rodents (PK-120-1; Laboratorsnab, Moscow, Russia) and to water.

The OXYS strain was derived from the Wistar strain of rats at the Institute of Cytology and Genetics, as described earlier [22]. The OXYS strain of rats was created by selection and inbreeding of Wistar rats sensitive to the cataractogenic effect of galactose. In the first five generations, cataract development was caused by a galactose-enriched diet; further selection was based on the spontaneous early development of a cataract, in conjunction with which the animals inherited the accelerated senescence syndrome manifesting itself as early development of age-related diseases, including retinopathy similar to human AMD.

To evaluate age-related changes in the JNK signaling pathway activity, we used male senescence-accelerated OXYS rats at preclinical (20 days), early (3 months), and late (18 months) stages of the AMD-like pathology (eight per group) and age-matched male Wistar rats. All experimental procedures were in compliance with the European Communities Council Directive of 24 November 1986 (86/609/EEC). The animal study protocol was approved by the Commission on Bioethics of the Siberian State Medical University (Protocol No. 4 008/4/06/2022, dated 20 June 2022). Every effort was made to minimize the number of animals used and their discomfort. IQ-1S–treated and untreated OXYS rats and Wistar rats were euthanized by an overdose of isoflurane inhalation anesthesia; the posterior wall of both eyes was carefully excised.

### 2.2. Treatment of Animals

IQ-1S was administered daily to 4.5-to-6.0-month-old Wistar and OXYS rats of the experimental groups (*n* = 15 in each group) via a gastric tube at a dose of 50 mg/kg of body weight per day as a suspension in 2 mL of a 1% starch solution. IQ-1S (series M314) was synthesized in the Department of Biotechnology and Organic Chemistry at Tomsk Polytechnic University (Tomsk, Russia). Control Wistar and OXYS rats received the vehicle only (1% starch solution). The animals were euthanized by CO_2_ inhalation.

### 2.3. Western Blotting

Retinas from untreated Wistar (control) and OXYS rats aged 20 days, 3 months, and 18 months and from 4.5-to-6.0-month-old Wistar and OXYS rats treated with either vehicle or IQ-1S (*n* = 5 per group) were homogenized in RIPA lysis buffer supplemented with protease and phosphatase inhibitors (Sigma-Aldrich, Saint Louis, MO, USA). Total protein concentration was determined by the BCA assay (Thermo Fisher Scientific, Waltham, MA, USA). Proteins were separated by electrophoresis in a 12% polyacrylamide gel and transferred to a nitrocellulose membrane (Bio-Rad, Hercules, CA, USA), which was then blocked with 5% bovine serum albumin in 10 mM phosphate buffer (pH 7.4) for 1 h. Next, the membrane was incubated at 4 °C overnight with primary antibodies, such as antibodies against JNK3, phospho-JNK3 (Thr183 + Tyr185) (ZB10H12 and PA5-36753, Invitrogen, Carlsbad, CA, USA), phospho-c-Jun (Ser63), phospho-APP (Thr688), and β-actin (AF3089, AF3084, and BF0198; Affinity Biosciences, Shanghai, China; 1:1000), and synapsin 1 (ab64581; Abcam, Waltham, MA, USA; dilution, 1:1000), and then for 1 h with a secondary anti-rabbit or anti-mouse antibody (ab6721 and ab97046; Abcam, Waltham, MA, USA; dilution: 1:5000). The intensity of luminescence was evaluated using a ChemiDoc MP imaging system (Bio-Rad, Hercules, CA, USA) and ImageJ 1.x software (NIH, Bethesda, MD, USA).

### 2.4. Electron-Microscopic Examination

Retinal samples from Wistar rats, OXYS rats, and OXYS rats treated with IQ-1S (*n* = 10 per group) were fixed with 2.5% glutaraldehyde in 0.1 M sodium cacodylate buffer (pH 7.2) for 1 h, washed with 0.1 M sodium cacodylate buffer, and postfixed with 1% osmium tetroxide in the same buffer for 1 h. After that, the samples were washed with water and incubated in a 1% aqueous solution of uranyl acetate in the dark at room temperature for 1 h. The tissue samples were then dehydrated in a graded series of ethanol and acetone mixtures, and embedded into a mixture of epon-araldite resins. First, semithin sections, 1 μm thick, were obtained on an ultratome and stained with toluidine blue, then ultrathin sections were made. The latter were stained with uranyl acetate and lead citrate, and then examined under a transmission electron microscope (JEM 100 SX; Jeol, Tokyo, Japan) at the Multi-Access Center for Microscopic Analysis of Biological Objects, Institute of Cytology and Genetics, Siberian State University, a department of the Russian Academy of Sciences.

For quantitative analysis, electron-transparent regions were identified in the electron micrographs of synapses of the retina (15 photos per animal) at a standard magnification of 30,000×. We determined the number of interneuronal contacts (per visual field of 100 μm^2^) and calculated numerical density of synapses per 100 μm^2^. Asymmetrical and symmetrical synapses were also counted. Numerical density of symmetrical and asymmetrical flat synapses (with straight profiles in cross-sections), positively curved synapses (bending toward the presynaptic part), and negatively curved synapses (bending toward the postsynaptic part) was calculated next. The length of the active contact zone (ACZ) was determined in ImageJ version 1.53e (National Institutes of Health, Bethesda, MD, USA). The synapses were classified (by the length of the ACZ) into small (<300 nm), medium-sized (300–500 nm), large (500–700 nm), and very large (>700 nm).

### 2.5. Histological Examination

For histological and immunohistochemical assays, the tissue samples (*n* = 10 per group) were fixed in 10% neutral formaldehyde in 0.1 M phosphate buffer (pH 7.4). Serial frontal sections (4 to 5 µm thick) were prepared, stained with hematoxylin and eosin, and examined under a photomicroscope (Axiostar Plus, Carl Zeiss, Oberkochen, Germany). Morphometric parameters were measured by quantitative image analysis performed in the Axiovision software, version 4.8 (Zeiss, Thornwood, NY, USA). The evaluation was performed through examination of five sections of the retina from each animal at a magnification of 10× and 100×, using a field of view of 900 μm^2^. The percentage of ganglionic neurons with total and focal chromatolysis was determined, as was the number of ganglionic and association neurons with nuclear pyknosis per 200 corresponding cells of the layer.

### 2.6. Immunohistochemical Examination

Paraffin blocks of retinal samples from Wistar rats, OXYS rats, and OXYS rats (*n* = 10 per group) treated with IQ-1S were sectioned by means of a microtome at a thickness of 5 µm. Glass slides with sections containing intact tissue were placed in a thermostat at 60 °C for 30 min incubation. The sections were then deparaffinized with subsequent rehydration. Next, high-temperature antigen retrieval was performed in citrate buffer (0.01 M; pH 6.0). Blocking of endogenous peroxidase, incubation with a primary antibody (anti-synapsin-1; CUSABIO, Houston, TX, USA; dilution, 1:300), incubation with a secondary antibody (included in the visualization system kit), and subsequent staining were performed using the Peroxidase Mouse & Rabbit Kit (DAB Liquid; 100 tests) visualization system (Diagnostic BioSystems, Pleasanton, CA, USA), according to the manufacturer’s protocol, with a negative control involving an immunohistochemical reaction on retinal sections not incubated with the primary antibody, and a positive control involving the Wistar rat brain tissue. The results of the immunohistochemical reaction were considered positive when a reddish-brown coloration was detected in the cytoplasm or on the plasma membrane of the cells under study. All 10 rats of each group were used, and synapsin 1 expression was assessed by means of the intensity and prevalence of staining.

### 2.7. Statistical Analysis

This analysis was performed using the STATISTICA 10.0 software package (Statsoft, Tulsa, OK, USA) and factorial analysis of variance (ANOVA) with a post hoc comparison of group means (Newman–Keuls test). The nonparametric Mann–Whitney test was carried out to assess the significance of differences in the comparison of mean values. The differences were considered statistically significant at *p* < 0.05.

## 3. Results

### 3.1. Changes in Levels of JNK3, APP, and c-Jun Phosphorylation in the Retina of Wistar and OXYS Rats with Age

**Figure 1:**
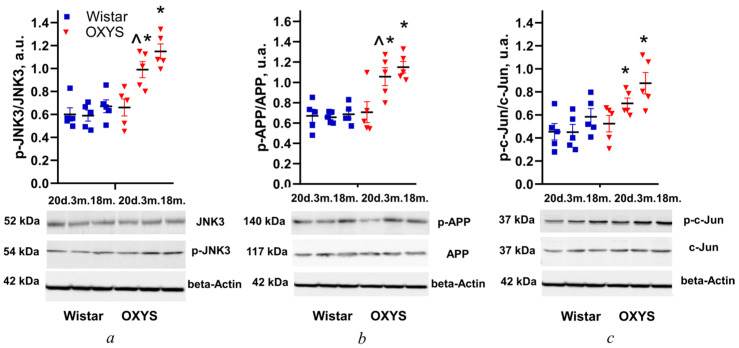
Effects of age on the levels of JNK3 (**a**), APP (**b**), and c-Jun (**c**) phosphorylation (a phospho-protein’s amount/the protein’s total amount) in the retina of Wistar and OXYS rats at 20 days, 3 months, and 18 months of age. Beta-Actin was used as the control. The data are presented as mean ± SEM (*n* = 5); ^ *p* < 0.05: a significant difference from rats of a previous age of the same strain; * *p* < 0.05: a significant difference between Wistar and OXYS rats of the same age.

The level of JNK3 phosphorylation was evaluated based on the p-JNK3/JNK3 ratio in retinas of Wistar and OXYS rats at ages of 20 days and 3 and 18 months. According to the Western blotting, the ratio depended on the animals’ genotype (strain) (F_1,24_ = 37.7; *p* < 0.001), and changed with age (F_2,24_ = 10.1; *p* < 0.001). The level of JNK3 phosphorylation at the age of 20 days was not different between Wistar and OXYS rats, but then it rose in OXYS rats with age. At ages of 3 and 18 months, this parameter in OXYS rats was higher than that in Wistar rats (*p* < 0.002 and 0.001, respectively; Figure 1a).

According to ANOVA, the level of APP phosphorylation (p-APP/APP) in Wistar and OXYS rat retinas depended on the animals’ genotype (F_1,24_ = 28.3; *p* < 0.001), and changed with age (F_2,24_ = 5.9; *p* < 0.008). A comparison of mean values among the analyzed groups indicated that at the age of 20 days, the phosphorylation levels in the retina did not differ between Wistar and OXYS rats (*p* > 0.05). APP phosphorylation in Wistar rats remained at the same level from 20 days to 18 months of age, whereas in OXYS rats it had increased by 3 months of age (*p* < 0.033) and at 3 and 18 months was higher than that in Wistar rats (*p* < 0.002 and 0.001, respectively; Figure 1b).

According to the ANOVA, the level of c-Jun phosphorylation (p-c-Jun/c-Jun) in the rat retinas was affected by the factors “genotype” (F_1,24_ = 12.2; *p* < 0.002) and “age” (F_2,24_ = 5.8; *p* < 0.009). The level was not different between the two rat strains at age 20 days (Figure 1c). By the age of 3 months in OXYS rats, it had increased to the trend level (*p* = 0.070) and was higher than that in Wistar rats (*p* < 0.016), just as at the age of 18 months (*p* < 0.038; Figure 1c).

### 3.2. IQ-1S Prevents the Increase in Levels of JNK3, APP, and c-Jun Phosphorylation in the Rat Retina

**Figure 2:**
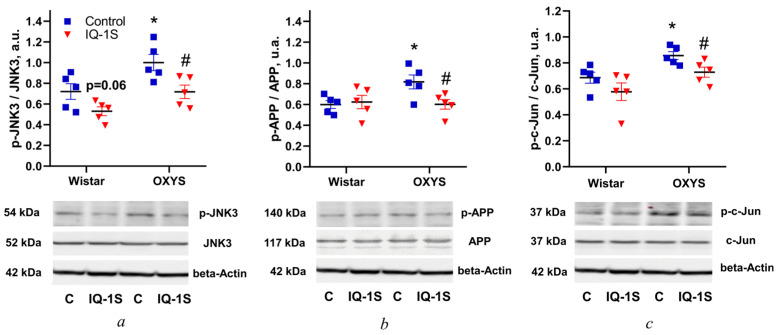
Effects of IQ-1S on levels of JNK3, APP, and c-Jun phosphorylation in the retina of 4.5-to-6.0-month-old Wistar and OXYS rats. Representative Western blot images and ratios p-JNK3/JNK3 (**a**), p-APP/APP (**b**), and p-c-Jun/p-c-Jun (**c**) in the retina of Wistar and OXYS rats treated with either vehicle or IQ-1S. β-Actin served as a loading control. The data are presented as mean ± SEM (*n* = 5); * *p* < 0.05: a significant difference between Wistar and OXYS rats of the same age; ^#^ *p* < 0.05: a significant effect of IQ-1S administration.

As expected, in the vehicle-treated control groups, the ratio of p-JNK to JNK3 at the age of 6 months was higher in OXYS rats than in Wistar rats (*p* < 0.033; Figure 2a). IQ-1S significantly diminished the ratio of p-JNK to JNK3 in the retina of OXYS rats (*p* < 0.023), but in Wistar rats the drug did so only marginally significantly (*p* = 0.060; Figure 2a).

The level of APP phosphorylation (p-APP/APP) in the retina of 6-month-old control OXYS rats was higher than that in control Wistar rats (*p* < 0.022; Figure 2b), whereas administration of IQ-1S reduced this ratio, but only in OXYS rats (*p* < 0.029; Figure 2b). The ratio of p-c-Jun to c-Jun in the retina of control OXYS rats was higher than that in Wistar rats (*p* < 0.012; Figure 2b). IQ-1S administration decreased this parameter only in OXYS rats (*p* < 0.033).

### 3.3. IQ-1S Attenuates Neurodegeneration in the Ganglion Layer and Associated Synaptic Disorders in the Inner Plexiform Layer of OXYS Rats

**Figure 3:**
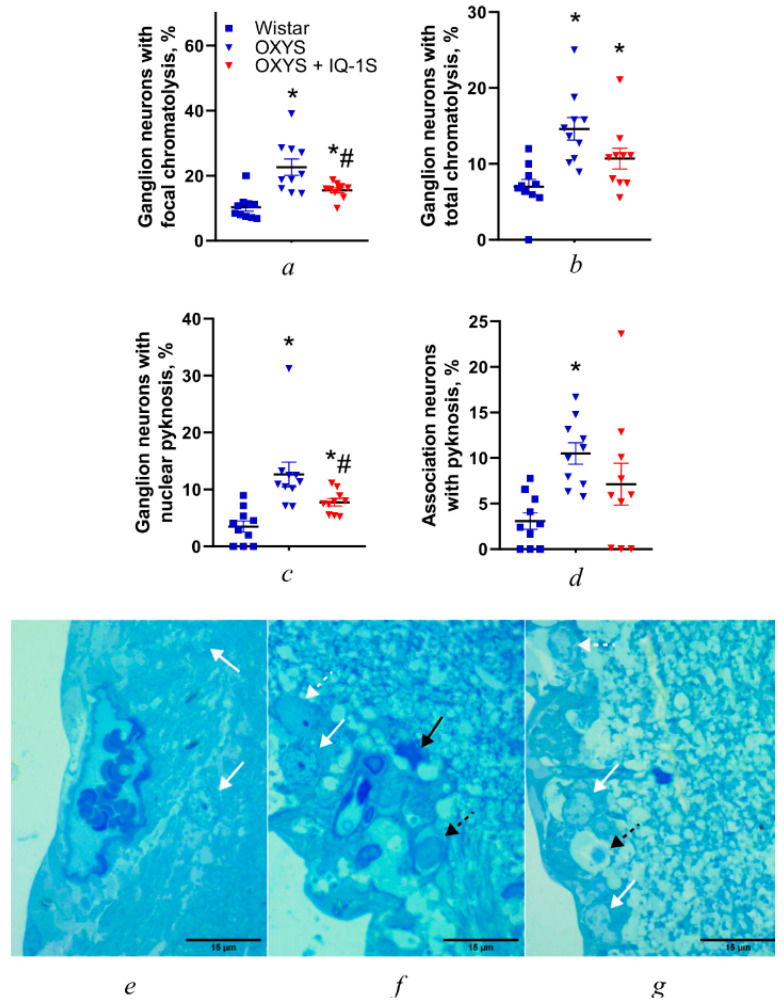
Effects of IQ-1S (at 50 mg per day from 4.5 to 6.0 months of age) on morphometric parameters of the retina in OXYS rats: ganglion neurons with focal chromatolysis (**a**), ganglion neurons with total chromatolysis (**b**), ganglion neurons with nuclear psychosis (**c**), and association neurons with pyknosis (**d**). The data are presented as mean ± SEM (*n* = 10); * *p* < 0.05: a significant difference from the Wistar strain; ^#^ *p* < 0.05: a significant effect of IQ-1S administration. Representative images of retinal ganglion layers of Wistar rats (**e**), OXYS rats (**f**), and OXYS rats treated with IQ-1S (**g**) (staining with toluidine blue). The neurons of Wistar rats (**e**) show a large number of Nissl granules (white arrows), whereas the neurons of OXYS rats show pronounced focal (white dotted arrow) and total chromatolysis (black dotted arrow), as well as neurons with pyknosis of the nucleus (black arrow). The course of IQ-1S administration limited degenerative changes: most neurons had intact Nissl granules (white arrows), and the damage was predominantly focal (white dotted arrow). Magnification: 1000×. Scale bars = 15 µm.

In this study, we confirmed that at the age of 6 months, the main morphological signs of AMD-like pathology are well-pronounced in all the layers of OXYS rats’ retinas, as reported earlier [20]. Here, we showed that the proportion of neurons with signs of degenerative changes in the retina of OXYS rats is significantly higher than that in Wistar rats (Figure 3), owing to an increase in the number of cells with signs of focal and total chromatolysis and in the number of neurons with pyknosis (*p* < 0.001, *p* < 0.001, *p* < 0.001, and *p* < 0.001, respectively; Figure 3a–d). Thus, neurodegeneration manifests itself not only as neuronal death, but also as a decrease in their volume, accompanied by a decline of the amount of the Nissl substance in the cytoplasm (chromatolysis) and a decrease in the size of the nucleus, in conjunction with chromatin condensation. Total chromatolysis can affect either a part of the cell (focal chromatolysis) or its entire volume with the transformation of neurons into a “ghost form,” which indicates their death. Its extreme manifestation is pyknosis of the nucleus: irreversible chromatin condensation, denoting cell death [23].

The proportion of ganglionic neurons with signs of focal chromatolysis declined slightly during IQ-1S administration (*p* = 0.028; Figure 3a). The small volume of perikaryon cytoplasm and of organelles in it were characteristic of neurons of the inner nuclear layer of the retina. Therefore, during assessment of their condition, only neurons with signs of pyknosis of the nucleus were counted. The proportion of association neurons with pyknosis of the nucleus in the inner nuclear layer of OXYS rats was three times greater than that in Wistar rats (*p* < 0.001), and IQ-1S did not significantly affect it (Figure 3d–g).

Toluidine blue staining revealed an elevated level of chromatophilic Nissl substance destruction in ganglion neurons of the retina of OXYS rats compared to Wistar rats (Figure 3e,f). This finding confirms our previously obtained data on damage to organelles and vacuolization of the cytoplasm in these neurons [21], and may indicate dysfunction of the intracellular infrastructure of protein synthesis [24].

Treatment with IQ-1S significantly reduced the proportion of ganglionic neurons with signs of pyknosis (*p* < 0.015; Figure 3c,g) approximately 1.5-fold, but it remained higher than that in Wistar rats.

**Figure 4:**
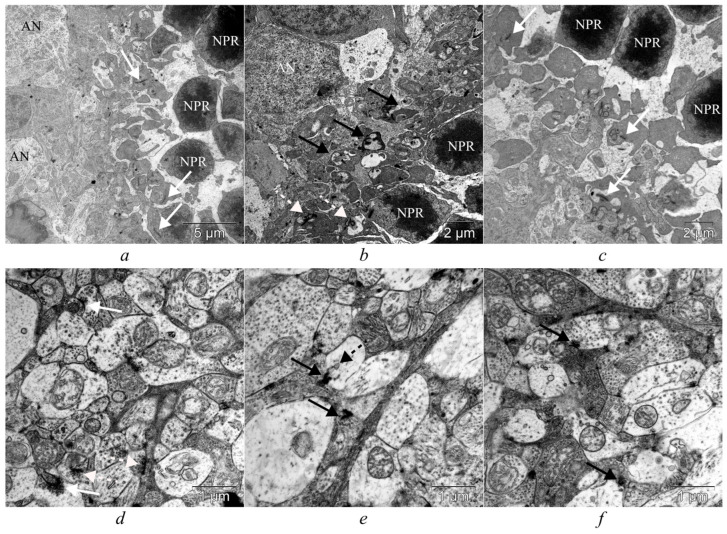
IQ-1S treatment improved the ultrastructure of the OPL of the retina in 6-month-old OXYS rats. Ultrastructural analysis of the retina of Wistar rats (**a**), OXYS rats (**b**), and IQ-1S–treated OXYS rats (**c**). NPR: photoreceptor cell nucleus, AN: association neuron. White arrows: synaptic ribbons of normal structure, white dotted arrows: osmiophilia, disruption of the correct structure of synaptic ribbons of OXYS rats, black arrows: degenerative changes in neuronal processes of the dark type. In the retina from Wistar rats (**d**), positively curved synapses (white arrows) and flat synapses (white dotted arrows) have normal ultrastructure, with synaptic vesicles not aggregated. In both OXYS (**e**) and OXYS+IQ-1S (**f**) rats, presynaptic terminals show light-type changes (edema), agglutination of synaptic vesicles (black arrows), and emergence of lysosomes in presynaptic terminals (black dotted arrow), but in OXYS+IQ-1S rats the degenerative alterations are less pronounced.

Retinal synapses are of two types: conventional synapses and specialized ribbon synapses. Conventional synapses, which consist of an active zone containing synaptic vesicles in the presynaptic terminal and a postsynaptic density in the postsynaptic terminal, are formed by all retinal cell types, except for photoreceptors and bipolar cells. Photoreceptors and bipolar cells form ribbon synapses, and vary in their functional properties, depending on the cell type [25,26]. As our electron-microscopic examination revealed, at the age of 6 months, the outer plexiform layer in OXYS rats was thinner than that in Wistar rats; there were processes of neurons of the dark type with osmiophilia of the cytoplasm, disintegration of mitochondria, and vacuolization (Figure 4).

Unlike the retina of Wistar rats, in the inner plexiform layer of OXYS rats degenerative changes in synaptic contacts of the light (edematous) type were observed. These changes are characterized by swelling of presynaptic terminals, their vacuolization, disintegration of mitochondria, agglutination of synaptic vesicles, and the appearance of lysosomes in synaptic terminals (Figure 4a,b). Morphological and ultrastructural changes in the synapses of the inner plexiform layer were nonspecific, and manifested themselves to various degrees of severity in both control and IQ-1S–treated OXYS rats; however, in the treated OXYS rats, the degenerative changes were less common (Figure 4a–f).

Given that the inner plexiform layer of the retina is involved in more complex and differentiated processing of visual information—including modulation and integration of signals to implement various aspects of vision such as movement, contrast, color, and depth—in this study, we decided to examine synapses specifically in this layer of the retina in more detail [27,28]. According to the current understanding of this topic, functionally mature synaptic contacts are synapses with asymmetric terminals (flat and positively and negatively curved), while symmetrical ones are the opposite: functionally immature perforated synapses are an intermediate stage in the formation of autonomous imperforate synapses, via splitting of actively functioning hypertrophied contacts. On the other hand, among functionally mature synapses, according to some authors, only synaptic contacts with a positively curved terminal should be considered active at this time point; these transition into a state of inhibition (as the pool of synaptic vesicles is consumed and conformational changes occur), i.e., become flat and negatively curved [29,30].

**Figure 5:**
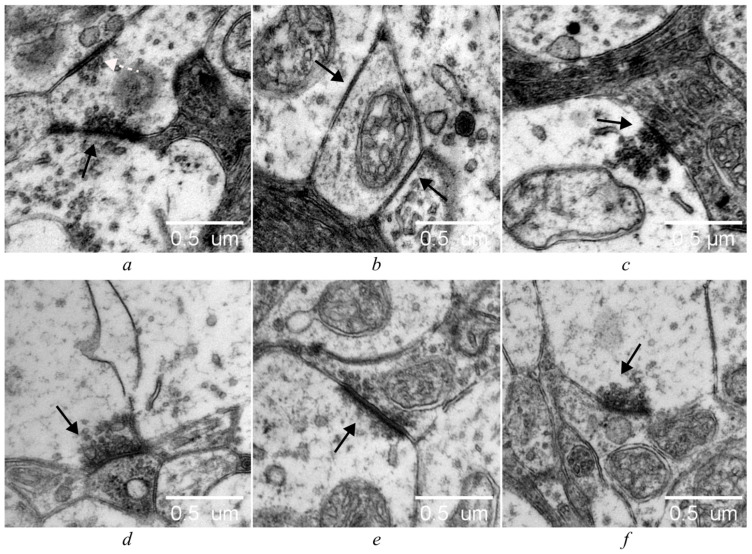
Ultrastructural analysis of different types of synapses in the inner plexiform layer of the rat retina. (**a**) A perforated synapse in the Wistar rat retina: a synaptic contact with two active zones (black arrow) and a flat synapse with a straight profile in the cross-section (white dotted arrow). (**b**) Symmetric synapses of an OXYS rat without a pronounced postsynaptic density (black arrows) and a flat synapse (**c**) with a straight profile in the cross-section (black arrow). Synapses of IQ-1S–treated OXYS rats: (**d**) a positively curved synapse of an IQ-1S–treated OXYS rat with a profile curved toward the presynaptic terminal (black arrow); (**e**) a flat synapse with a straight profile in the cross-section (black arrow); (**f**) a negatively curved synapse with a profile curved toward the postsynaptic membrane (black arrow). Scale bars: 500 nm.

In the present study, we quantified each type of synapse (Figure 5). The total density of synaptic contacts in the retina of OXYS rats was 1.5 times lower as compared to the retina of Wistar rats (*p* = 0.043); this decrease was due to degeneration of asymmetric synapses (*p* = 0.011; Figure 5). It should be pointed out that percentages of symmetric and asymmetric contacts among all synapses differed in each rat strain and amounted to ~35% and ~65%, respectively, in Wistar rats, and ~47.5% and ~52.5%, respectively, in OXYS rats. Treatment with IQ-1S increased the number of asymmetric contacts (*p* = 0.035; Figure 5; Supplementary Table S1).

**Figure 6:**
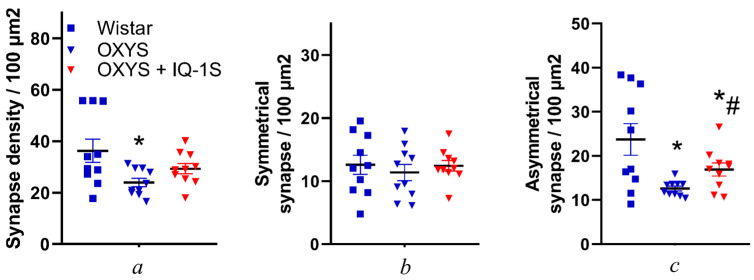
Ultrastructural analysis synapses of retinal ganglion cells in 6-month-old Wistar rats, OXYS rats, and OXYS rats with IQ-1S OXYS rats: synapse density of the retina (**a**), the proportion of symmetric (**b**) and asymmetric synapses (**c**) in the total synapse density. The data are presented as mean ± SEM (*n* = 10); * difference significant with Wistar; # *p* < 0.05: a significant effect of IQ-1S administration. IQ-1S was given at 50 mg per day from 4.5 to 6 months of age.

An important part of the synaptic contact is the area of the presynaptic membrane where a neurotransmitter is released into the synaptic cleft: the synapse active zone, which ensures the approach of synaptic vesicles to the presynaptic membrane and the release of their contents into the synaptic cleft. In percentage terms, rats of each group showed predominance of contacts with ACZ lengths of 100–200 and 200–300 nm: in Wistar rats, both these contacts accounted for ~27% of the total number of synapses, whereas in OXYS rats they accounted for ~29% (Figure 6). The proportion of contacts with ACZ lengths of 100–200 and 200–300 nm did not change during treatment with IQ-1S. The proportion of contacts with ACZ lengths of <100, 300–500, 500–700, and >700 nm did not differ between Wistar and OXYS rats. Treatment with IQ-1S contributed to a decrease in the proportion of contacts with ACZ lengths of 500–700 nm (*p* < 0.011; Supplementary Table S1) in OXYS rats.

### 3.4. IQ-1S Enhances Synapsin 1 Expression in the OXYS Rat Retina

**Figure 7:**
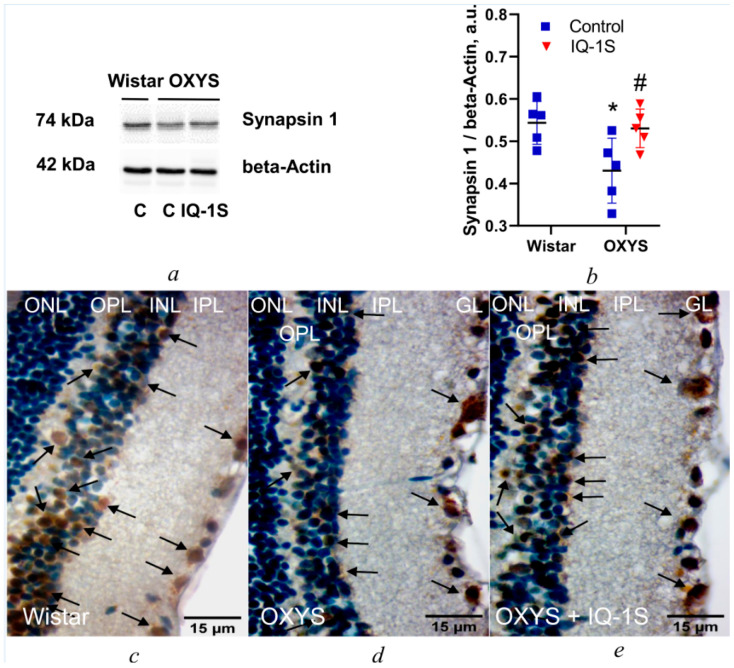
The expression of synapsin 1 in the retina of 6-month-old Wistar and OXYS rats and the effect of IQ-1S on it in the retina of OXYS rats. Representative Western blot images (**a**) and relative amounts of synapsin 1 (**b**) in the retina of Wistar and OXYS rats treated with vehicle and OXYS rats treated with IQ-1S. β-Actin served as a loading control. The data are presented as mean ± SEM (*n* = 5); * *p* < 0.05: a significant difference between Wistar and OXYS rats of the same age; ^#^ *p* < 0.05: a significant effect of IQ-1S administration. Representative images of the inner nuclear, inner plexiform, and ganglionic layers of the retina in Wistar rats (**c**), OXYS rats (**d**), and IQ-1S–treated OXYS rats (**e**); (*n* = 10); stained with the anti–synapsin-1 antibody and hematoxylin (Ehrlich). ONL: outer nuclear layer, OPL: outer plexiform layer, INL: inner nuclear layer, IPL: inner plexiform layer, and GL: ganglionic layer. Synapsin 1 in association neurons and ganglionic neurons and in the inner plexiform layer is indicated by black arrows. Magnification: 1000×. Scale bars = 15 µm.

To confirm our results, we assayed synapsin expression in the retina of the rat groups. In the vehicle-treated control groups, the level of synapsin 1 in the retina of 6-month-old OXYS rats was lower than that in Wistar rats of the same age (*p* < 0.025; Figure 7a,b). IQ-1S upregulated synapsin 1 in the retina of OXYS rats (*p* < 0.037; Figure 7a,b).

In addition, we performed immunostaining of retinas with the antibody to synapsin 1, a crucial protein in synaptogenesis and synaptic function [31]. Our results suggested that synapsin 1 was expressed in association neurons and inner plexiform and ganglion layers of the retina in animals of all groups (Figure 7c–e). In Wistar rats, high expression levels of this protein were found in each of the three retinal layers mentioned above (Figure 7c), whereas in OXYS rats, the intensity and prevalence of staining were significantly lower (Figure 7d). Treatment with IQ-1S significantly strengthened the expression of synapsin 1, judging by the intensity and prevalence of staining (Figure 7e).

## 4. Discussion

Growing evidence suggests that dysregulation of MAP kinases—including JNK, extracellular signal-regulated kinase 1 and 2 (ERK1/2), and p38—plays a crucial role in the pathogenesis of age-related neurodegenerative diseases [32]. Accordingly, control of MAPK signaling pathways has long been discussed as a potential therapeutic target in neurodegenerative diseases, including AMD [8,33,34]; however, there have been no data on JNK signaling in the retina and its changes with aging and with AMD development. Here, for the first time, we assessed JNK3 signaling activity in the retina and did not detect its age-related changes in healthy Wistar rats; we showed that manifestation and progression of the AMD-like pathology in OXYS rats occurs simultaneously with JNK pathway activation. We also confirmed that the selective JNK3 inhibitor IQ-1S is able to suppress neurodegenerative changes in the OXYS rat retina, and demonstrated that its effects are prevention of the destructive changes in retinal synapses and suppression of JNK3 signaling pathway activity during active progression of AMD signs in OXYS rats.

As we have reported earlier, by the age of 3 months and concurrently with alterations of RPE cells and of choroidal microcirculation, 100% of OXYS rats develop clinical signs of retinopathy corresponding to the dry type of AMD in humans. Significant pathological changes in the RPE and clinical signs of advanced stages of retinopathy are evident in OXYS rats at age ~12 months [21]. These changes manifest themselves as excessive accumulation of lipofuscin and amyloid in RPE regions [21] and disturbances in the morphology of the RPE sheet (including an increase in the proportion of multinucleated cells, hypertrophy, a distortion of cell shape, and reactive gliosis), and intensify with age. Accordingly, by the age of 6 months, the main morphological signs of the AMD-like pathology are well-pronounced in the retina of the OXYS strain, implying that the age from 4 to 6 months is a period of active progression of the disease [18].

Here, we assayed JNK signaling pathway activity in the retina, using as an objective indicator the phosphorylation level of the key kinase JNK3 and of its target proteins: c-Jun and APP. Using age-matched Wistar rats as a control, we examined OXYS rats’ retinas at the age of 20 days when clinical manifestations of AMD are absent, then in the period of their active manifestation at the age of 3 months, and at the age of 18 months, which is when neurodegenerative changes are pronounced. Unexpectedly, in contrast to the activity of ERK1/2 and p38 MAPK pathways [35,36], the JNK-signaling activity in the retina did not change with age in healthy aging Wistar rats, while manifestation and progression of the AMD-like pathology in OXYS rats occurred simultaneously with JNK pathway activation. p-JNK3 accumulation in the retina of OXYS rats was registered at the age of 3 and 18 months, in the period of manifestation and progression of the AMD-like pathology. Its key manifestation is degenerative changes in RPE cells and the death of neurons, implying a connection between the development of the AMD-like pathology in OXYS rats and the activation of the JNK signaling pathway.

The progression of AMD is accompanied by impaired proteostasis in the retina with deposition of pathological aggregates in the form of drusen [37]. Just as in amyloid plaques in the brain during AD, the composition of the drusen includes aggregates of amyloid β. Between the two diseases, the detrimental intra- and extracellular deposits have many similarities [38]. Previously, we have discovered a disturbance of proteostasis with the accumulation of pathological β-amyloid and hyperphosphorylated tau protein during the development of AMD in the retina of OXYS rat [35,36]. Here, APP was employed as a target protein that determines the activity of the JNK signaling pathway. We found that the magnitude of its phosphorylation in the OXYS rat retina is elevated in the same age periods as the amount of p-JNK3, thus confirming the activation of this signaling pathway. Additionally, we noticed hyperphosphorylation of a target protein of the JNK signaling pathway (c-Jun) in the retina of OXYS rats. Thus, we demonstrated that developing neurodegenerative changes in the retina in OXYS rats by the age of 3 months occur simultaneously with activation of the JNK signaling pathway in the retina.

Here, we showed that IQ-1S treatment prevents the age-related enhancement of JNK3 phosphorylation in the retina of OXYS rats, and reduces c-Jun phosphorylation: possible evidence of suppression of JNK signaling pathway activity. APP served as a target protein of the JNK signaling pathway to assess the effect of IQ-1S administration on its activity. IQ-1S slowed down JNK3-dependent phosphorylation of APP in the retina of OXYS rats. Our data are consistent with results of an experiment conducted earlier, in which IQ-1S administration suppressed neuronal death in the brain of OXYS rats [18], in conjunction with a decline in the activity of the JNK signaling pathway [19].

Degenerative changes of neurons during aging and neurodegenerative diseases are accompanied by the loss of interneuronal synaptic contacts. Synaptic transmission, essential for visual phenomena in the brain, declines with age, owing to age-related changes in the neuroretina, and these alterations may be potentiated by AMD risk factors, leading to the induction and progression of this disease [39].

At early stages of neurodegeneration, this problem manifests itself as synaptic dysfunction, which can be reversible. As the process progresses, however, damage to synapses and spines becomes irreversible, thereby ultimately causing the loss of both synapses and neurons [40]. JNK affects the synaptic dysfunction in vivo and in vitro in AD models. For instance, in an AD animal model, JNK inhibition strongly attenuates the synaptic impairment while also ameliorating the cognitive deficits [3]. Nonetheless, we failed to find any studies on the relation between JNK signaling-pathway activity and synaptic function in retinal neurons.

In this study, we observed pronounced degenerative changes in retinal neurons of 6-month-old OXYS rats. According to light microscopy, they manifested pronounced chromatolysis, whereas at the ultrastructural level we saw disintegration of the granular endoplasmic reticulum, of the Golgi complex, and of mitochondria, as well as vacuolization of the cytoplasm and signs of pronounced degenerative changes in the synapses. Because we have previously shown [19], and here confirmed, that neurodegenerative changes are already present in the retina of 6-month-old OXYS rats, the destructive changes in synapses were an expected finding.

We noted a decrease in overall numerical density of synapses and in the proportion of functionally mature synaptic contacts. Disruption of protein synthesis inevitably leads to damage to neuronal cytoskeleton structure, thereby contributing to disorganization of synaptic vesicle transport; together with the observed downregulation of synapsin 1 in retinal neurons, this effect can give rise to synaptic-vesicle aggregates in presynaptic terminals of OXYS rat neurons. Aggregation of synaptic vesicles and shortening of the active zone of synaptic contacts point to impairment of synaptic transmission. The above transformations of cyto- and synaptic architecture of the retina may reflect accelerated aging and can indicate disturbances of synaptogenesis processes and plastic reorganization of interneuronal connections [41]. Such remodeling of the cyto- and synaptic architectonic organization will inevitably be accompanied by a change in the arrangement of neuronal processes. This arrangement determines potential synaptic partners from which a neuron can choose, and as a consequence, a change in the integrative-triggering activity of neurons and in the nature of the spread of information within the neural network; these alterations create preconditions for a change in the integration of signals in a neural circuit and can eventually negatively affect visual function [3,42].

As we have reported previously [17,19], neuroprotective properties of IQ-1S are associated with its ability to normalize rheological properties, to improve blood circulation, to increase the functional activity of the RPE, to reduce VEGF expression in endothelial cells, and to upregulate PEDF in the neuroretina. It has also been shown previously that IQ-1S attenuates the degeneration of photoreceptors and neurons of inner layers, significantly improves retinal ultrastructure, and increases the number of mitochondria, which is significantly lower in the neuroretina of OXYS rats compared to Wistar rats. The previously obtained results were confirmed when we assessed the state of synaptic contacts here: the suppression of the JNK signaling pathway by IQ-1S limited the degenerative changes in functionally mature synaptic contacts, while causing a significant increase in the number of synapses with an ACZ length of 100–200 nm, concurrently with a decrease in destructive changes in the synthetic apparatus of neurons. These data indicate an improvement in synaptic signal transmission, the activation of neosynaptogenesis processes, or the reorganization of larger contacts; that is, the engagement of adaptive mechanisms that prevent visual impairment during aging. This beneficial phenomenon probably provides prerequisites for gradual restoration of normal synapse structure and of signal transmission within the neural circuit of the retina in senescence-accelerated OXYS rats. Thus, the results of the present study confirm that JNK signaling is a promising therapeutic target in AMD and that IQ-1S can be a good prophylactic strategy against this disease.

## 5. Conclusions

The JNK3 signaling activity in the retina of healthy Wistar rats did not change with age, in contrast to OXYS rats, in which manifestation and progression of the AMD-like pathology occurred simultaneously with JNK pathway activation. We confirmed here that the selective JNK3 inhibitor IQ-1S is able to suppress neurodegenerative changes in the OXYS rat retina and showed that its effects are prevention of the destructive changes in retinal synapses and the suppression of the JNK3 signaling pathway activity during active progression of AMD signs in OXYS rats.

## Data Availability

All data necessary to reproduce the results are contained within the article. Raw data are available upon request.

## Supplementary Materials

The following supporting information can be downloaded at: https://www.mdpi.com/article/10.3390/cells14120896/s1, Table S1: Effects of IQ-1S on quantitative synaptic parameters of retinal ganglion cells in OXYS rats.

## Abbreviations

ACZ	Active contact zone
AD	Alzheimer’s disease
AMD	Age-related macular degeneration
APP	Amyloid beta precursor protein
IQ-1S	Sodium salt of 11H-indeno[1,2-b]quinoxalin-11-one oxime
JNK	C-Jun N-terminal kinase
OPL	Outer plexiform layer
RPE	Retinal pigment epithelium
