# Supplementary material for: Alterations of JNK Signaling Pathway Activity in the Rat Retina: Effects of Age, Age-Related Macular Degeneration-like Pathology, and a JNK Inhibitor (IQ-1S)
Source: Cells. 2025 Jun 13;14(12):896. doi: 10.3390/cells14120896 (PMC12190811; doi:10.3390/cells14120896)
Supplement: Supplementary file 1 [file cells-14-00896-s001.zip › cells-3617221-supplementary.pdf]

**Supplementary Table S1.** Effects of IQ-1S on quantitative synaptic parameters of retinal ganglion cells in OXYS rats.

| | | Wistar | OXYS | OXYS IQ-1S |
|---|---|---|---|---|
| Total synapse density | | 36.29 ± 1.70 | 23.94 ± 1.64*<br>p < 0,006 | 29.32 ± 2.01<br>#p = 0,052 |
| Perforated | | 0.16 ± 0.06 | 0.09 ± 0.06 | 0.59 ± 0.17*# |
| Symmetrical | | 12.57 ± 0.84 | 11.35 ± 0.79 | 12.41 ± 0.83 |
| Asymmetrical | Total | 23.72 ± 3.58 | 12.60 ± 0.54*<br>p < 0,002 | 16.91 ± 1.47*#<br>*p<0,002<br>#p=0,042 |
| | Flat | 11.12 ± 1.33 | 7.17 ± 0.47*<br>p < 0,005 | 7.85 ± 0.67*<br>p = 0,016 |
| | "+" curved | 8.58 ± 1.76 | 3.59 ± 0.36*<br>p = 0,005 | 6.20 ± 0.91#<br>p=0,016 |
| | "-" curved | 3.86 ± 0.38 | 1.75 ± 0.21*<br>P=0,016 | 2.27 ± 0.25* |
| Synapses with different ACZ lengths, % | <100 | 13.26 ± 2.03 | 14.66 ± 2.51 | 9.12 ± 1.89* |
| | 100-200 | 25.07 ± 2.43 | 28.80 ± 2.91 | 33.79 ± 2.63*#<br>*p = 0,012;<br>#p = 0,045 |
| | 200-300 | 25.27 ± 2.04 | 28.14 ± 2.90 | 30.17 ± 2.36 |
| | 300-500 | 18.48 ± 1.63 | 17.58 ± 2.40 | 14.17 ± 1.90 |
| | 500-700 | 8.68 ± 1.25 | 5.06 ± 0.93*<br>p = 0,010 | 5.23 ± 1.10*<br>p = 0,010 |
| | >700 | 4.57 ± 0.90 | 3.76 ± 1.02 | 4.19 ± 1.20 |

Note: The data are presented as mean ± SEM (n = 10); * difference significant between Wistar and OXYS rats; #p < 0.05: a significant effect of IQ-1S administration. IQ-1S was given at 50 mg per day from 4.5 to 6 months of age.
